# A Workflow to Investigate Exposure and Pharmacokinetic Influences on High-Throughput *in Vitro* Chemical Screening Based on Adverse Outcome Pathways

**DOI:** 10.1289/ehp.1409450

**Published:** 2015-05-15

**Authors:** Martin B. Phillips, Jeremy A. Leonard, Christopher M. Grulke, Daniel T. Chang, Stephen W. Edwards, Raina Brooks, Michael-Rock Goldsmith, Hisham El-Masri, Yu-Mei Tan

**Affiliations:** 1 Oak Ridge Institute for Science and Education, Oak Ridge, Tennessee, USA; 2 Lockheed Martin, Research Triangle Park, North Carolina, USA; 3 Chemical Computing Group Inc., Montreal, Quebec, Canada; 4 National Health and Environmental Effects Research Laboratory, U.S. Environmental Protection Agency, Research Triangle Park, North Carolina, USA; 5 Department of Epidemiology, University of Alabama at Birmingham, Birmingham, Alabama, USA; 6 National Exposure Research Laboratory, U.S. Environmental Protection Agency, Research Triangle Park, North Carolina, USA

## Abstract

**Background:**

Adverse outcome pathways (AOPs) link adverse effects in individuals or populations to a molecular initiating event (MIE) that can be quantified using *in vitro* methods. Practical application of AOPs in chemical-specific risk assessment requires incorporation of knowledge on exposure, along with absorption, distribution, metabolism, and excretion (ADME) properties of chemicals.

**Objectives:**

We developed a conceptual workflow to examine exposure and ADME properties in relation to an MIE. The utility of this workflow was evaluated using a previously established AOP, acetylcholinesterase (AChE) inhibition.

**Methods:**

Thirty chemicals found to inhibit human AChE in the ToxCast™ assay were examined with respect to their exposure, absorption potential, and ability to cross the blood–brain barrier (BBB). Structures of active chemicals were compared against structures of 1,029 inactive chemicals to detect possible parent compounds that might have active metabolites.

**Results:**

Application of the workflow screened 10 “low-priority” chemicals of 30 active chemicals. Fifty-two of the 1,029 inactive chemicals exhibited a similarity threshold of ≥ 75% with their nearest active neighbors. Of these 52 compounds, 30 were excluded due to poor absorption or distribution. The remaining 22 compounds may inhibit AChE *in vivo* either directly or as a result of metabolic activation.

**Conclusions:**

The incorporation of exposure and ADME properties into the conceptual workflow eliminated 10 “low-priority” chemicals that may otherwise have undergone additional, resource-consuming analyses. Our workflow also increased confidence in interpretation of *in vitro* results by identifying possible “false negatives.”

**Citation:**

Phillips MB, Leonard JA, Grulke CM, Chang DT, Edwards SW, Brooks R, Goldsmith MR, El-Masri H, Tan YM. 2016. A workflow to investigate exposure and pharmacokinetic influences on high-throughput *in vitro* chemical screening based on adverse outcome pathways. Environ Health Perspect 124:53–60; http://dx.doi.org/10.1289/ehp.1409450

## Introduction

The adverse outcome pathway (AOP) is a conceptual framework originally developed with the goal of utilizing pathways-based data to support ecotoxicology research and risk assessment (Ankley et al. 2010). Researchers in a variety of disciplines have since used AOPs to describe impacts of a chemical on molecular targets and biochemical pathways in a sequential manner (Lapenna et al. 2012; Vinken et al. 2013; Watanabe et al. 2011). The AOP framework begins with a molecular initiating event (MIE), which is defined as the interaction between a xenobiotic and a specific biomolecule (Ankley et al. 2010), such as inhibition of an enzyme due to competitive binding of a chemical in the active site (Russom et al. 2014). The MIE is followed by a progression of a defined series of key events (KEs) that are measurable through *in vitro* or *in vivo* assays, necessary for the development of the toxicological outcome, and connected by key event relationships (KERs). These KEs and KERs then lead to an apical outcome that is relevant to regulatory purposes (Villeneuve et al. 2014). Such outcomes may be survival, development, and reproduction at the population level in ecotoxicology; or disease and organ dysfunction in human individuals.

The power of the AOP framework arises from the knowledge that multiple chemicals can act through common biochemical pathways. Because there are tens of thousands of chemicals in commerce (Egeghy et al. 2012; U.S. EPA 2014b), starting from these common pathways provides a more rapid and cost-effective alternative for hazard screening compared with chemical-by-chemical approaches. Rather than relying on traditional toxicity tests conducted for individual chemicals (e.g., costly assays administered one at a time in animals), the AOP framework can support the use of high-throughput *in vitro* assays to quickly measure the activity of numerous chemicals with respect to a given molecular target. The AOP itself is chemical independent to allow for a general interpretation of results based on common modes of action and biological pathways. Practical application of AOPs in chemical-based risk assessment, however, will require extrapolation of an *in vitro* concentration expected to trigger an MIE to an *in vivo* biologically effective target tissue dose, which can then be used to estimate a regulatory-relevant external dose (i.e., using reverse toxicokinetics). This extrapolation cannot be made without considering exposure, as well as the absorption, distribution, metabolism, and excretion (ADME) properties of a chemical (Groh et al. 2015). The most active chemical in an *in vitro* assay may not induce *in vivo* toxicity if concentrations necessary to trigger an MIE are unlikely to be attained due to limited exposure or ADME-mediated processes.

To augment the application of an AOP framework in chemical risk assessment, we developed a workflow to incorporate exposure and ADME considerations for refining outcomes from *in vitro* assays designed based on an MIE. We evaluated the utility of this workflow using *in vitro* assay results from the ToxCast™ data set for a previously established AOP, acetylcholinesterase (AChE) inhibition (Russom et al. 2014). First, the identities of the active chemicals in the human AChE inhibition assay were obtained from the ToxCast™ data set (U.S. EPA 2012a). Next, the likelihood of these active chemicals to trigger an MIE in the brain was determined by sequentially considering their exposure potential, absorption potential, and ability to cross the blood–brain barrier (BBB) to bind to brain AChE. In addition, structural similarities of active chemicals were compared against structures of inactive chemicals using molecular fingerprint models to detect possible nonactive parents that might become biologically active after undergoing metabolism. This case study demonstrates the ongoing need for a more holistic approach that encompasses various considerations for improving toxicity predictions based on *in vitro* measurements and for expanding the AOP framework to improve its utility in chemical-specific risk assessment.

## Methods

**Figure 1:**
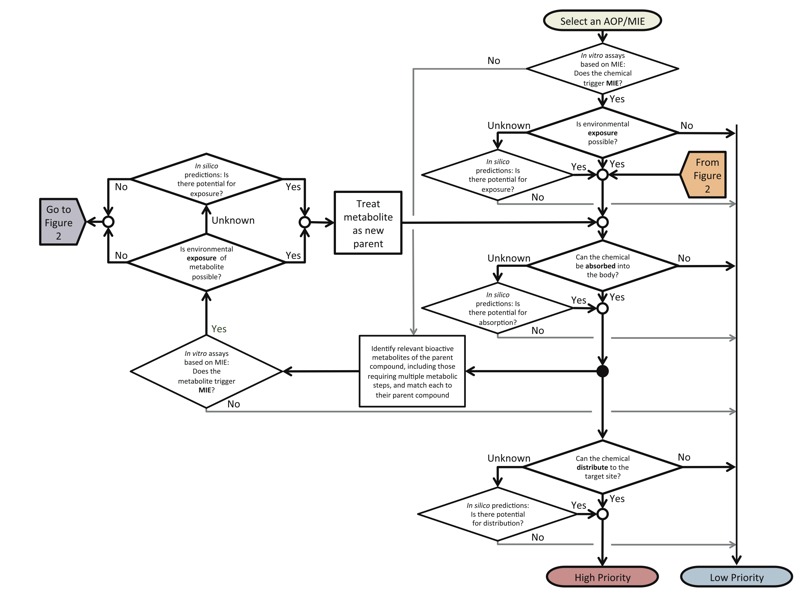
Workflow for including exposure and ADME considerations into the AOP framework. The chemical of interest is a parent compound. Exposure, absorption, distribution, and metabolism are considered for the parent compound, and distribution of a known metabolite of an identified parent compound (described in Figure 2) is considered if the parent exhibits exposure and absorption potential. Each step is evaluated based on available data. When insignificant, the chemical is classified as “low priority.” If any step results in an unknown effect, further research is needed (i.e., high-throughput follow-up studies). “High-priority” chemicals should be further ranked according to relationships among rates of absorption or distribution, activating or detoxifying metabolic processes, and excretion from a biological system. Open circles represent converging steps in the workflow, and solid black circles represent diverging steps.

**Figure 2:**
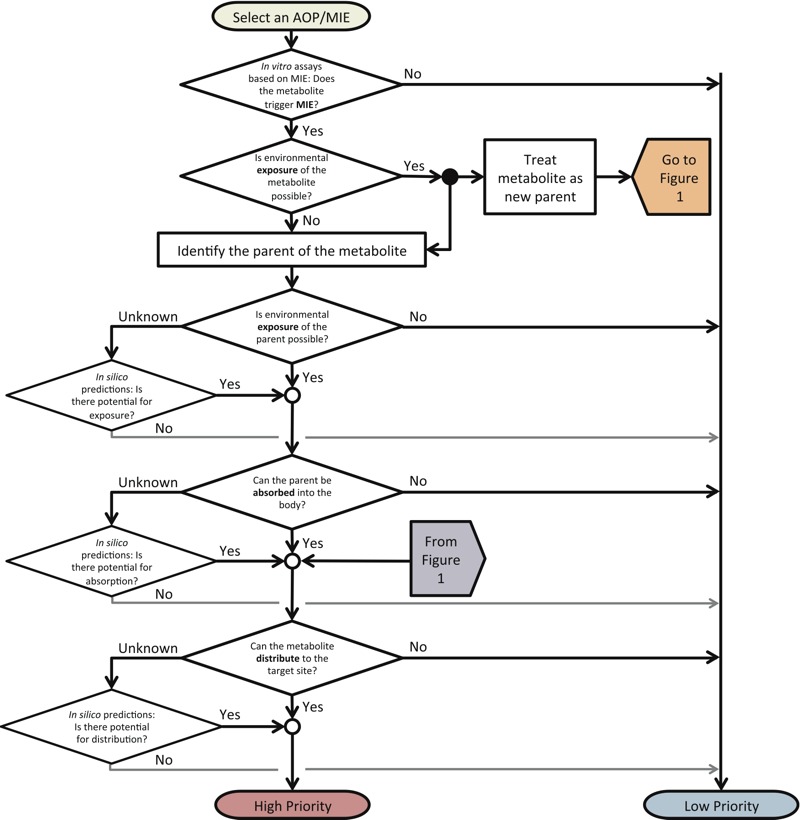
Workflow for including exposure and ADME considerations into the AOP framework. Exposure of the known metabolite is examined, and if exposure is possible the metabolite is then treated similar to a parent compound (described in Figure 1). If exposure of the metabolite is not possible, then its distribution is considered only if its identified parent exhibits exposure and absorption potential. Each step is evaluated based on available data. When insignificant, the chemical is classified as “low priority.” If any step results in an unknown effect, further research is needed (i.e., high-throughput follow-up studies). “High priority” chemicals should be further ranked according to relationships among rates of absorption or distribution, activating or detoxifying metabolic processes, and excretion from a biological system. Open circles represent converging steps in the workflow, and solid black circles represent diverging steps.

*Conceptual structure of the exposure–ADME workflow.* The exposure–ADME workflow incorporates exposure and ADME considerations for linking chemical exposure with AOP activation through the MIE. The main utility of this workflow is to refine *in vitro* results, which can then be used to predict *in vivo* MIEs that would trigger an AOP. This workflow begins with the selection of an AOP of interest, such as one listed in the AOP Wiki (https://aopkb.org/aopwiki/index.php/AOP_List). Next, active chemicals identified in a specific *in vitro* assay are examined as parent compounds (Figure 1) or metabolites (Figure 2). Given that these are “known” metabolites, it is assumed that *a*) they would be generated in the human body after exposure to their parent compounds, and *b*) the identity of their parent compounds is known. If the metabolite tests positive *in vitro*, its parent’s exposure and absorption potentials are examined, along with its own capability of reaching the molecular target (Figure 2). If the active metabolite can also be found in the environment, its own exposure potential and ADME-related properties are also examined (Figure 1).

*Exposure.* Each active chemical can be placed into one of three categories based on its exposure potential: widespread exposure, limited exposure (e.g., occupational exposures or patient exposures to specific drugs), or low/no potential of exposure (e.g., drugs that have failed clinical trials). Those chemicals with low/no exposure potential are considered “low priority.” The remaining chemicals are advanced to the next step of the workflow.

*Absorption.* Next, the physicochemical properties of the chemicals (e.g., lipophilicity or water solubility) are measured to assess absorption potential and bioavailability as related to primary routes of exposure. Many of these properties can be estimated using a combination of generalized molecular-based methods, such as geometric optimization and pharmacophore modeling (Goldsmith et al. 2012). A number of public or commercial platforms can also be used to estimate such properties through specific ADME-related molecular descriptors based on reference two-dimensional (2D) and three-dimensional (3D) chemical structures. Some of these tools and resources include ChemSpider (http://www.chemspider.com), QikProp (http://www.schrodinger.com/QikProp/), RDKit (http://www.rdkit.org), Dragon 6 (Mauri et al. 2006), and Chemistry Development Kit (Steinbeck et al. 2003).

*Distribution.* The likelihood that a chemical could be sequestered in certain tissues (e.g., fat or bone), bind to plasma proteins, and so on is assessed similarly using physicochemical properties. Chemicals that may be systematically distributed might require evaluation to see if they can access the molecular target. For example, if the target involves the central nervous system, a chemical must cross the BBB before binding. Chemicals not easily absorbed or distributed are considered “low priority.” The remaining chemicals are classified as “high priority,” as are those chemicals for which the data are insufficient to confidently assign “low-priority” status. When exposure, absorption, or distribution potential is uncertain, *in silico* approaches may be applied to generate estimates for guiding the prioritization process.

*Metabolism.* When a parent compound tests negative in an *in vitro* assay, it is still possible that its metabolite could reach a molecular target after the parent is absorbed into the body. Thus, our workflow suggests that known metabolites of inactive parents be subjected to *in vitro* testing (Figure 1). Predicting the likelihood of a compound being metabolized, as well as predicting the structures of its major metabolites, is challenging (Bertz and Granneman 1997; Shlomi et al. 2008). In the best-case scenario, *in vivo* testing is used to confirm any predictions, and confirmed metabolites can then be subjected to *in vitro* toxicity testing (Figure 2). Unfortunately, this scenario requires a substantial investment of time and resources. Alternatively, computational programs [e.g., Meteor Nexus (Lhasa Limited)] or *in vitro* metabolism assays may be used to predict metabolites based on enzymatic activity. Similarity analyses may then be used to identify which predicted metabolites have structures similar to known active chemicals, giving a preliminary indication that they might interact with the molecular target in a manner sufficient to trigger an MIE.

The previous steps of the workflow primarily address qualitative aspects of exposure and ADME to identify “high-priority” chemicals for additional quantitative analyses. An example of quantitative analysis is generating surrogates for exposure and ADME behaviors based on chemical properties (e.g., predicted biological half-life may be used to extrapolate clearance rate, and a faster clearance rate could be interpreted as lower availability of the chemical to the molecular target). The “high-priority” chemicals, identified from the qualitative evaluation, can then be ranked based on comparisons among measured or predicted intake doses, as well as relative rates of absorption/distribution and metabolism/excretion.

*ToxCast™ background and AChE assay results.* ToxCast™ is a multiyear effort led by the U.S. Environmental Protection Agency (EPA) to test thousands of chemicals in hundreds of assays, including enzyme inhibition assays (U.S. EPA 2014a). To date, > 2,000 chemicals have been tested in > 700 *in vitro* assays covering approximately 300 signaling pathways (U.S. EPA 2014a). Chemicals considered within ToxCast™ include, but are not limited to, additives, pesticides and antimicrobial agents, plasticizers, and pharmaceuticals that are in various stages of clinical testing or have been introduced into the commercial market. A full inventory of the chemicals used in the ToxCast™ program, of which the 1,059 considered in this study is a subset, is available online (http://www.epa.gov/ncct/dsstox/sdf_toxcst.html), with more chemicals to be added in the future. Detailed information regarding analytical quality control of procured ToxCast™ chemicals is provided in Supplemental Material, “Chemical quality control.”

The Novascreen acetylcholinesterase (AChE) analysis in ToxCast™ consists of an *in vitro* cell-free biochemical assay that detects the inhibition of human-derived AChE enzyme, as determined colorimetrically by enzyme reporter activity using the substrate acetylcholine and a positive control of physostigmine (U.S. EPA 2014a). Additional details of the assay procedures can be found in Supplemental Material, “Chemical assays.” Thirty of the 1,059 chemicals tested in the AChE inhibition assay were found to be active (3%).

*Prioritization of active chemicals in AChE inhibition assay.* The inhibition of AChE by parent compounds and known metabolites was considered to comprise the MIE (the first step in Figures 1 and 2). Literature collected from PubMed, PubChem, Web of Science, and technical documents was used to categorize exposure potential of the 30 active chemicals (Table 1). Data collected included primary route of exposure, chemical use category and history, prevalence of usage across the general population, and documented adverse health effects. Chemicals with low/no exposure potential were designated “low priority.”

**Table 1:** Inhibition activity (in decreasing order), exposure probability, and ADME properties of thirty compounds extracted from ToxCast™ data set identified as acetylcholinesterase inhibitors.

Compound	AC_50_ (μM)	Exposure^*a *^	Absorption^*b *^	Distribution^*c *^	Priority	Metabolism^*d *^	Source
Chlorpyrifos oxon	0.149	1	Yes	Yes	High	+	Eaton et al. 2008; Smegal 2000
PharmaGSID_47259	0.287	4	NA	NA	Low	–	U.S. EPA 2010
Carbofuran	0.416	3	Yes	Yes	High	±	Hussain et al. 1990; U.S. EPA 2008
Anthralin	0.512	2	Yes	No	Low	–	McGill et al. 2005
Naled	1.01	1	Yes	Yes	High	–	Duprey et al. 2008; U.S. EPA 2006
Carbosulfan	1.21	1	Limited	Yes	High	±	Abass et al. 2010
Raloxifene hydrochloride	1.85	2	Limited	No	Low	–	Kosaka et al. 2011
1-Benzylquinolinium chloride	2.48	2	Yes	Yes	High	U	U.S. EPA 2012b
Besonprodil	3.49	3	Yes	Yes	High	–	Ouattara et al. 2009
Bendiocarb	4.09	3	Yes	Yes	High	–	Berman et al. 2011, 2012
SB236057A	4.63	4	NA	NA	Low	–	Roberts et al. 2001
GW473178E	4.79	4	NA	NA	Low	–	U.S. EPA 2010
SSR241586	4.86	3	Limited	Yes	High	–	Métro et al. 2011
SSR69071	5.05	4	NA	NA	Low	–	Kapui et al. 2003
Mevinphos	5.11	3	Yes	Yes	High	±	Cochran et al. 1996; U.S. EPA 1994
Azamethiphos	6.6	2	Yes	Yes	High	±	EMEA 1999
Oxamyl	7.4	2	Yes	Yes	High	–	EXTOXNET 1993; Schilmann et al. 2010
Gentian violet	7.65	1	Yes	Yes	High	+	TOXNET 2013
Toluene-2,4-diisocyanate	8.78	2	Yes	Yes	High	±	U.S. EPA 2013
Didecyldimethylammonium chloride	12.1	1	Limited	Yes	High	–	Dejobert et al. 1997; Houtappel et al. 2008
Propoxur	12.7	1	Yes	Yes	High	±	Ostrea et al. 2014
Methomyl	13.9	3	Yes	Yes	High	–	EXTOXNET 1996; Van Scoy et al. 2013
Pentamidine isethionate	16.8	2	Limited	No	Low	NA	Beach et al. 1999; Montgomery et al. 1990
bis(2-Ethylhexyl) decandioate	17	4	NA	NA	Low	=	NIOSH 1983
SR125047	17.6	4	NA	NA	Low	–	Kohlhaas et al. 2006
PharmaGSID_48172	18.3	4	NA	NA	Low	–	U.S. EPA 2010
Dodecylbenzenesulfonic acid	19.3	1	Limited	Yes	High	±	TOXNET 2002
SSR150106	20.9	3	Yes	Yes	High	+	R & D Focus Drug News 2007
Mercuric chloride	23.1	2	Yes	Yes	High	+	Bernhoft 2012; Boscolo et al. 2009
Bronopol	23.3	1	Yes	Yes	High	±	Cui et al. 2011; Travassos et al. 2011
Abbreviations: AC_50_, concentration of chemical necessary to reduce maximum activity of the AChE enzyme by 50%; ADME, absorption, distribution, metabolism, and excretion; BBB, blood–brain barrier; NA, not applicable. ^***a***^Exposure conditions are as follows: 1, widespread exposure to public; 2, occupational-only or special cases of exposure; 3, unknown exposure; 4, low likelihood of exposure (no further analysis). ^***b***^Absorption considers whether chemicals are free of violations (“Yes”) of the “Rule of 5” code (Lipinski et al. 1997); properties that violate these rules include large size and molecular weight, high number of rotatable bonds, and an excessive number of hydrogen bond donors or hydrogen bond acceptors. ^***c***^Distribution considers whether chemicals can cross (“Yes”) the BBB. ^***d***^Metabolism is considered to be transformation to an active metabolite (+), detoxification (–), possibility of activation or detoxification (±), metabolite with same toxicity as parent (=), parent excreted with no metabolism (NA), or unknown (U).							

When comprehensive review resulted in greater confidence that a chemical (or the parent of a tested metabolite) would exhibit widespread or limited exposure, its potential for absorption into the body was queried using the ADMET Predictor™ (Simulations Plus Inc.). These chemicals’ 2D simplified molecular-input line-entry system (SMILES) structures were entered into the ADMET Predictor™ to estimate their physicochemical properties, such as water solubility, octanol-water partition coefficient (log *K_ow_*), plasma protein binding, p*K_a_*, and skin permeability. Chemicals with negligible absorption were designated as “low priority.”

Next, BBB permeability (i.e., distribution to the molecular target) was queried using the ADMET Predictor™ for the remainder of the chemicals (those not considered “low priority”), as well as inactive chemicals structurally similar to the original 30 active chemicals. Molecular structures of these chemicals were washed of extraneous salts, had protonation states rebalanced, had explicit hydrogen atoms augmented, and had their energy states minimized through conversion into a 3D conformation using Molecular Operating Environment (MOE) software (Chemical Computing Group) before being entered as a predictive data set in the ADMET Predictor™. The chemical space of SMILES structures of washed and energy-minimized chemicals was compared to that of the ADMET Predictor™ S+BBB filter (a binary classifier of “high” or “low” permeability collected from 1,942 chemicals from multiple sources and with a classification concordance value of 93%). Chemicals deemed unable to cross the BBB were designated “low priority.” Chemicals with widespread or limited exposure, possible absorption into the body, and potential to reach brain AChE were designated as “high priority” candidates, which can be further ranked in the future based on their relative rates of absorption/distribution and metabolism/excretion.

*Similarity analysis.* We identified inactive chemicals falling within a related functional or structural class as active chemicals, along with parent compounds of known metabolites that exhibited a positive response in the AChE inhibition assay, by evaluating their structural similarities to such active chemicals. Similarity tests were conducted through use of MOE, in which molecular fingerprints were selected based on the presence or absence of one of the 166 public MDL Information Systems’ structural Molecular Access System (MACCS) keys (Willett et al. 1998). The fingerprint of each of the 30 active chemicals was used to identify the nearest neighbor inactive chemical using a Tanimoto similarity threshold of 75%, which is considered to be an appropriate cut-off value for fingerprint searches (Rahman et al. 2009). Briefly, the Tanimoto similarity threshold coefficient is the ratio of the number of bit-key characteristics common to both sample sets (the size of the intersection) divided by the number of bit-key characteristics found in either or both sets (the size of the union), and thus explains the similarity and diversity of the sample sets (Baldi and Nasr 2010).

## Results

The ToxCast™ human AChE assay had 30 active chemicals. Following the steps in our workflow, seven active chemicals were assigned as “low priority” due to a low likelihood of exposure to the general population or workers (Table 1). A majority of these “low-priority” chemicals were pharmaceuticals that had failed in clinical trials. Eight chemicals had a low likelihood of exposure to the general population, but might be of concern to workers who regularly come into contact with them or to individuals with special medical conditions that would require their use. Another 8 chemicals were considered as presenting a high exposure potential to the general public. The exposure potential of the 7 remaining chemicals was unknown. These chemicals included pesticides for which manufacture or distribution have been cancelled but may still be present in the environment or that may have derivatives that are still in use, as well as pharmaceuticals that may be cleared for public use after the later stages of clinical safety trials.

Most of the 23 chemicals with high/limited exposure potential were predicted to have significant oral absorption, followed by inhalation. Only anthralin and bendiocarb are expected to have greater dermal than oral absorption. Six chemicals were predicted to have barriers to absorption as a result of physicochemical properties such as excessive charge, high molecular weight, or a high degree of lipophilicity (Table 1). Two of these 6 chemicals—raloxifene hydrochloride and pentamidine isethionate—were also predicted to have low potential for BBB penetration. The third chemical with predicted inability to cross the BBB was anthralin (Table 1). The remaining 4 chemicals with limited absorption were retained as “high priority” chemicals but can be assigned a lower ranking in future quantitative analysis. Application of our workflow resulted in a total of 10 “low-priority” chemicals (7 due to low exposure potential and 3 due to low BBB permeability), leaving approximately 67% to be further analyzed.

Metabolism was shown to affect a chemical’s activity in several ways. It was an activating step for some chemicals such as the organophosphate (OP) pesticide chlorpyrifos (Table 1), which is metabolized to the most active chemical in the AChE assay, chlorpyrifos oxon. For other chemicals, metabolism was a detoxifying step (Table 1), as was the case for the OP pesticide naled, which is metabolized to the less potent chemical dichlorvos before being further metabolized and excreted from the body.

Fifty-two of the 1,029 inactive chemicals exhibited a similarity threshold > 75% with their nearest active neighbors. Twenty-nine chemicals were structurally similar to bis(2-ethylhexyl) decanedioate (a “low-priority” chemical), which has very limited absorption through the primary exposure routes of skin and lungs and little to no adverse toxicity upon incidental oral ingestion (NIOSH 1983). Although individuals in the general public may be exposed by using products containing this chemical, it would not be absorbed through the skin (Clayton et al. 1994). Thus, these 29 chemicals that are structurally similar to bis(2-ethylhexyl) decanedioate were also designated as “low priority.” Zamifenacin (an M3 selective muscarinic antagonist) demonstrated 76% similarity with the poorly absorbed and distributed “low-priority” chemical raloxifene hydrochloride, so it was also given a “low-priority” status, leaving 22 chemicals remaining on the list of “possible false negatives” (see Supplemental Material, Table S1, for similarity scores for these 22 chemicals). The elimination of 30 chemicals using our prescreening approach allowed focus on the more relevant “false negatives.”

Examples of these “false negatives” of interest included chlorpyrifos, which showed 91% similarity with its oxon metabolite; dichlorvos, which showed 83% similarity with its parent naled; and aldicarb, which showed 88% similarity with the carbamate methomyl (see Supplemental Material, Table S1). At least two of these inactive chemicals—chlorpyrifos and chlorpyrifos methyl—were parents of active metabolites likely to inhibit AChE. In addition, at least four of these inactive chemicals—aldicarb, trichlorfon, dichlorvos, and phosalone—were themselves known as moderate to weak AChE inhibitors *in vivo*.

## Discussion

In an AOP framework, known adverse outcomes may be linked via KEs to an upstream MIE. Knowledge of an MIE can be used to design high-throughput *in vitro* assays to screen for chemicals able to trigger that MIE. However, exposure potential and ADME properties that may influence a chemical’s ability to reach a molecular target *in vivo* are rarely considered beyond *in vitro* outcomes. In the present study we attempted to address this issue through the development of a conceptual workflow that provides general guidance for considering exposure and ADME when interpreting *in vitro* results. The utility of this workflow was demonstrated using the active chemicals from the human AChE inhibition assay in the ToxCast™ data set. Ten of 30 chemicals were designated as “low priority” from our analyses: 7 had very low exposure potential, and 3 were unlikely to cross the BBB. Those remaining were designated as “high priority” and can be subjected to future ranking based on quantitative considerations of ADME.

The value added by considering exposure and ADME properties of a chemical to refine high-throughput *in vitro* results can be illustrated using the top five active chemicals in the ToxCast™ assay. PharmaGSID_47259 is a failed pharmaceutical for which little information is available to the public. Because of a high likelihood of no exposure potential, it was designated as “low priority.” Anthralin is a topical medication approved for treating psoriasis; it is sequestered in the mitochondria of keratinocytes, where it triggers apoptosis and promotes the growth of new skin tissue (Sehgal et al. 2014). Due to its lipophilicity and sequestration in dermal tissues, anthralin is unlikely to enter the systemic circulation to a significant extent and is also predicted to have low BBB permeability, resulting in it being a “low-priority” chemical. The other three chemicals—carbofuran, naled, and chlorpyrifos oxon—are known AChE inhibitors. Carbofuran is a metabolite of carbosulfan, another highly reactive compound, and is itself metabolized to the equally toxic form 3-hydroxycarbofuran through hydroxylation, or to the less toxic moiety 3-ketocarbofuran through oxidation (Gupta 1994). Naled is metabolized to dichlorvos or to the nontoxic chemicals dimethyl phosphate and bromodichloroacetaldehyde (Roberts and Hutson 1999). The most interesting case, however, involves chlorpyrifos oxon.

Chlorpyrifos oxon is a chief metabolite of chlorpyrifos after enzymatic activity occurs within the body, and humans are exposed to trace amounts of oxon directly as chlorpyrifos is environmentally degraded (Mackay et al. 2014). It is well-established that AChE inhibition is the AOP arising from chlorpyrifos exposure (U.S. EPA 2011). Chlorpyrifos itself is not an AChE inhibitor, as shown in both *in vivo* and *in vitro* studies (Chambers and Carr 1993). From an *in vivo* perspective, chlorpyrifos can be considered a “false negative” as a result of its inability to demonstrate reactivity in the AChE *in vitro* assay, but its metabolite exhibits potent activity. This chemical highlights the critical need to consider ADME, especially metabolism, in establishing a more holistic interpretation of high-throughput *in vitro* results based on AOPs. In our study, structural similarities of inactive and active chemicals were compared in order to detect “true *in vitro* negatives, but false *in vivo* negatives,” such as chlorpyrifos, that might become biologically active when accounting for metabolism.

The carbamate pesticide aldicarb is a known AChE inhibitor *in vivo* (Wyld et al. 1992) but is considered to be inactive in the AChE inhibition assay. From our analysis, it was identified as a possible false negative because it was structurally similar to methomyl (88%). Although aldicarb was banned in the United States in 2010, distribution from the manufacturing company is not expected to be completely eliminated until 2017 (Cone 2010), suggesting that exposure to the population remains possible until then. Two other inactive chemicals identified in similarity analysis were the OPs trichlorphon (86% similar to naled) and phosalone (81% similar to azamethiphos). Trichlorphon and phosalone are considered moderate to weak AChE inhibitors *in vivo* and represent other examples of “possible false negatives.”

In a prior analysis of 309 ToxCast™ chemicals, 14 were considered AChE inhibitors in both rat and human *in vitro* assays (Knudsen et al. 2011). Eight of these chemicals were included in our current “high-priority” list, and the 6 remaining chemicals were very weak human AChE inhibitors, 2 of which, aldicarb and dichlorvos, were identified as possible “false negatives” from our similarity analysis at a threshold of 75%. If this threshold was further decreased to 60%, an additional 3 chemicals would be selected, leaving only malaoxon (56% similarity with mevinphos) unidentified from our analysis. The comparison of our results with those of a previously published study demonstrates the usefulness of similarity analysis in detecting possible “false negatives” from *in vitro* data. A logical next step to enhance the utility of our workflow would be to compare these possible “*in vivo* false negatives” with *in silico* predictions [e.g., quantitative structure–activity relationship (QSAR) or protein-docking models] that are built to identify initiation of MIEs by chemical classes rather than by individual chemicals. *In vitro* assays and *in silico* models are complementary approaches able to incorporate high-throughput analyses into AOP frameworks.

There is often some difficulty in the extrapolation of *in vitro* and *in silico* results to *in vivo* observations due to a variety of factors. Sometimes, *in silico* predictions contradict *in vitro* results, and further evaluation of the appropriateness of either approach is necessary. A possibility exists that the fundamental assumptions in either or both approaches are inadequate. For example, the use of 2D descriptors alone in predictive *in silico* QSAR models may lack the required specificity to account for protein–ligand interactions observed in homochiral protein environments (Chang et al. 2012; Vedani and Smiesko 2009). Domain of applicability issues may also arise for chemicals that fall outside the chemical descriptor space that was used to build QSAR models (Dragos et al. 2009; Jaworska et al. 2005; Stanforth et al. 2007; Tan et al. 2012). Identification of factors responsible for interassay variability is necessary to also avoid interpretation errors of *in vitro* results (Beresford et al. 2000; LeBlanc et al. 2011). Human error or, more often, promiscuous chemicals that can bind or interfere with assay reagents and targets may lead to incorrect conclusions derived from *in vitro* results. Such problems were discovered by Baell and Walters (2014) when applying *in vitro* testing to drug development, and similar problems are likely to exist with environmental chemicals as well. For example, pan-assay interference compounds (PAINS) can show signs of activity in assays due to redox cycling, degradation, or other nonspecific processes that lead to a signal, even when not truly binding to a molecular target’s active site (Baell and Walters 2014). In the present study, the SMILES strings of the 20 high priority chemicals were entered into the open-source BioActivity Data Associative Promiscuity Pattern Learning Engine (BADAPPLE plugin) (UNM 2014) and into MOE to evaluate the promiscuity of these chemicals. Both programs yielded high promiscuity scores for gentian violet, likely due to interference of the dye with assay absorption spectra (Baell and Holloway 2010), as well as for the fracking agent 1-benzylquinolinium. Although 1-benzylquinolinium may be toxic, it is unlikely to specifically inhibit AChE. Recognition of promiscuous compounds allowed for further reduction in our list of “high-priority” chemicals.

Finally, when both *in silico* and *in vitro* methods suggest the same outcome, consideration of ADME-mediated behaviors of chemicals continues to be important, because it is difficult for either approach to depict the complexity of biological processes. Much like the concept of prodrugs (Rautio et al. 2008), an inactive parent may become active through metabolic processes, or a compound considered active may not reach its molecular target as a result of limited absorption or rapid clearance. Therefore, our workflow can be used to examine outcomes predicted by *in silico* and *in vitro* approaches.

Excretion, although a critical component of ADME, was addressed only briefly in the present study because we investigated only qualitative aspects of ADME in detail. Qualitative consideration of excretion alone does not sufficiently predict the ability of a chemical to reach its molecular target. Rather, it is the rate of excretion compared with the rate of absorption that will determine whether a chemical can bind with its molecular target at a concentration sufficient to trigger an MIE. Thus, rate of excretion, along with rates of metabolism, absorption, and distribution will be addressed in a future work to illustrate the quantitative aspects of our workflow.

## Conclusions

The importance of incorporating exposure and ADME properties in refining results of high-throughput *in vitro* assays designed based on an MIE was demonstrated through our developed workflow. Twenty of 30 possible active chemicals identified in a human AChE inhibition assay were prioritized for future quantitative testing. Similarity analysis allowed 22 inactive chemicals from the *in vitro* assay to be identified as possible “false negatives.” Some of these chemicals are either parents of potential active metabolites or weak AChE inhibitors *in vivo*. Our workflow improves the reliability of *in vitro* testing by identifying false negatives (e.g., inactive parents of active metabolites) and reduces cost and time by screening out false positives (e.g., active chemicals with no exposure potential) that may otherwise have undergone unnecessary analyses.

## Supplemental Material

(129 KB) PDF
