# Supplementary material for: A Workflow to Investigate Exposure and Pharmacokinetic Influences on High-Throughput in Vitro Chemical Screening Based on Adverse Outcome Pathways
Source: Environ Health Perspect. 2015 May 15;124(1):53–60. doi: 10.1289/ehp.1409450 (PMC4710605; doi:10.1289/ehp.1409450)
Supplement: (129 KB) PDF [file ehp.1409450.s001.acco.pdf]

Environ Health Perspect
DOI:10.1289/ehp.1409450

Note to Readers: *EHP* strives to ensure that all journal content is accessible to all readers. However, some figures and Supplemental Material published in *EHP* articles may not conform to 508 standards due to the complexity of the information being presented. If you need assistance accessing journal content, please contact ehp508@niehs.nih.gov. Our staff will work with you to assess and meet your accessibility needs within 3 working days.

Supplemental Material

A Workflow to Investigate Exposure and Pharmacokinetic Influences on High-Throughput *in Vitro* Chemical Screening Based on Adverse Outcome Pathways

Martin B. Phillips, Jeremy A. Leonard, Christopher M. Grulke, Daniel T. Chang, Stephen W. Edwards, Raina Brooks, Michael-Rock Goldsmith, Hisham El-Masri, and Yu-Mei Tan

Table of Contents

Methods for ToxCast^{TM} protocol

Chemical quality control

Chemical assays

References

Table S1. Structural similarities between "high priority" active chemicals and twenty-two remaining nearest neighbor inactive chemicals as determined through the presence or absence of structural Molecular Access System (MACCS) keys.

Methods for ToxCast™ protocol

Chemical quality control

Chemical information associated with the ToxCast library (MSDS, CAS registry numbers, molecular weight, handling procedures, etc.) were quality reviewed before being entered into the ChemInventory database and structure-annotated into US EPA's DSSTox Project http://www.epa.gov/ncct/dsstox/. DSSTox chemical identifiers (GSID, chemical name, CAS registry number, etc.) were then entered into the ChemInventory Bottle and Sample ID table to establish linkage between the ChemInventory and DSSTox Master databases. See http://www.epa.gov/ncct/dsstox/ChemicalInfQAProcedures.html for detailed information regarding chemical information quality review.

Chemicals procured through suppliers were also subject to analytical procedures to check purity, identity, and concentration. Chemicals were diluted in dimethyl sulfoxide (DMSO) to a final concentration of 3 mM in 20 μL and analyzed by OpAns Analytical Laboratory on an LC/MS (liquid chromatography mass spectrometry) platform. Those failing identity (molecular weight) checks with purity less than 50% were subject to additional LC/MS testing, as well as GC/MS (gas chromatography mass spectrometry) analysis at the National Institutes of Standards and Technology. Additional information regarding analytical QC of procured chemicals can be found at http://www.epa.gov/ncct/toxcast/chemicals.html.

Chemical assays

The Novascreen acetylcholinesterase (AChE) analysis, developed and run by Caliper, a PerkinElmer company, consisted of an *in vitro* cell-free biochemical assay that detected the inhibition of human-derived AChE enzyme as determined colorimetrically by enzyme reporter activity using the substrate acetylcholine and a positive control of physostigmine (eserine).

ToxCast™ Phase I and Phase II chemicals were diluted using 20 mM DMSO and added to 96-well plates at a single concentration of 10 or 25 μM. If a chemical exhibited a significant response (enzyme inhibition), then a concentration-response approach was used.

Raw data (percent activity versus positive control) from each chemical analysis was received by EPA and processed within the custom workflow for all assays in the ToxCast™ project (Judson et al. 2012). Phase I and Phase II data were subjected to curve-fitting algorithms for computing and processing to determine potency (AC_{50} or concentration that elicited 50% activity) and efficacy (E_{max} or maximum response). A four-parameter Hill function was fit to the concentration-response curves using the *nls* package of the open source language "R" (R Development Core Team 2008) and included the following assumptions: concentration-responses were monotonic, outliers were removed when there was a monotonic curve, a variable slope was allowed, and negative inhibition (activation) was allowed for the enzymatic assay. Curve asymptotes were constrained to -10 and 10% (lower) and 100 and 110% (upper) to maintain consistent extrapolation of AC_{50} for each assay-compound combination, and extrapolations were allowed at a 3-fold lower concentration than the lowest tested concentration and a 3-fold higher concentration than the highest test concentration so that potential activity could be captured outside the normal test range. Additional criteria for determining AC_{50} included the R^2 coefficient of determination for Hill curves at ≥ 0.6, p values ≤ 0.01, and E_{max} values $\geq 30\%$ baseline activity. AC_{50} values were transformed as $-\log(AC_{50}/1000000)$ to reflect potency. A compound that was considered to be inactive in an assay was set to 1,000,000 μM (i.e., 1M) before transformation to ensure a potency of 0.

References

Judson RS, Martin MT, Egeghy P, Gangwal S, Reif DM, Kothiya P, et al. 2012. Aggregating data for computational toxicology applications: The U.S. Environmental Protection Agency (EPA) Aggregated Computational Toxicology Resource (ACToR) System. Int J Mol Sci 13:1805-1831.

R Core Team. 2013. R: A language and environment for statistical computing. R Foundation for Statistical Computing, Vienna, Austria. Available: http://www.R-project.org/ [accessed 30 April 2015].

Table S1. Structural similarities between "high priority" active chemicals and twenty-two remaining nearest neighbor inactive chemicals as determined through the presence or absence of structural MACCS[a] keys.

Inactive Chemical	Active Chemical	Similarity Score
Sodium dodecylbenzenesulfonate	4-Dodecylbenzenesulfonic acid	100
Dodecylbenzene sulfonate triethanolamine	4-Dodecylbenzenesulfonic acid	100
Dodecyltrimethylammonium chloride	Didecyldimethylammonium chloride	96
Chlorpyrifos	Chlorpyrifos oxon	91
SR144190A	SSR241586	90
2-(Hydroxymethyl)-2-nitro-1,3-propanediol	Bronopol	89
Aldicarb	Methomyl	88
SAR102779	SSR241586	88
Trichlorfon	Naled	86
Dichlorvos	Naled	83
Phosalone	Azamethiphos	81
SSR146977	SSR241586	81
UK-343664	SSR69091	79
2,2′,4,4′-Tetrahydroxybenzophenone	Anthralin	78
Chlorpyrifos-methyl	Chlorpyrifos oxon	76
PharmaGSID_47258	SSR241586	76
SSR240612	SSR69071	76
N,*N*-Dimethyloctylamine	Didecyldimethylammonium chloride	75
N-Methyldioctylamine	Didecyldimethylammonium chloride	75
Volinanserin	SR125047	75
AVE3247	PharmaGSID_48172	75
SAR377142	SSR241586	75

[a]Molecular Access System
